# Tracing Taonga Trajectories: A Methodological Framework for Indigenous Heritage Mapping

**DOI:** 10.1002/snz2.70012

**Published:** 2026-01-18

**Authors:** Marina Ferrari de Aquino Klemm, Charlotte Milne, Isaac Brown, Sandi Ringham, Wendy A. Nelson

**Affiliations:** ^1^ Natural Sciences Department Auckland Museum Auckland New Zealand; ^2^ Institute for Resources, Environment and Sustainability University of British Columbia Vancouver Canada; ^3^ Te Ara Whānui Kaitaia New Zealand; ^4^ University of Waikato Hamilton New Zealand; ^5^ School of Biological Sciences University of Auckland Auckland New Zealand

**Keywords:** collection, colonization, data sovereignty, decolonization, museum, stocktake, voyages

## Abstract

Rangitāhua is a tupuna to Ngāti Kuri and represents the iwi's geographic and ancestral connection to the Pacific. Despite this millennium‐long ancestral tie, Ngāti Kuri's access to Rangitāhua has been severed for two centuries. Meanwhile, many European expeditions visited the islands, extracting and distributing natural history taonga across institutions, mostly in the Northern Hemisphere. In this context of disconnection, Ngāti Kuri engaged partners to reclaim research leadership over Rangitāhua, leading to the Indigenous‐led Te Mana o Rangitāhua program, embedding Māori values and tikanga within the environmental wellbeing research project. This study is part of the program and documents our collaborative approach to identifying expeditions to Rangitāhua, mapping where their taonga and data are held worldwide, and examining institutional responses to our data requests. We identified 127 expeditions that distributed 1.73 million objects across 88 institutions. Our provenance mapping successfully cross‐linked specimens to the expeditions that collected them and the institutions that house them today. However, our research also revealed ongoing institutional barriers to data access, emphasizing colonial gatekeeping practices embedded in contemporary museology systems. We stress the urgent need for accessible and reciprocal data request systems if museum practitioners hope to advance the ultimate goal of Indigenous data sovereignty.

## 1 Introduction

Rangitāhua is a chain of sixteen volcanic islands lying halfway between Te Moana‐a‐Toitehuatahi (Bay of Plenty) and Tonga, holding the title of Aotearoa's northernmost land masses and serving as an ancestral home and sentinel territorial marker of Ngāti Kuri. As mana whenua of Rangitāhua through historical occupation, Ngāti Kuri maintained cultural and spiritual connections to the islands for over a millennium, understanding and relating to them as a tupuna who connects both the iwi and Aotearoa culturally, geographically, and politically to the Pacific.

Rich kōrero describe Ngāti Kuri occupations and visits to Rangitāhua, including accounts of the ancient waka *Tāhirirangi* and *Taikoria*, as well as the *Kurahaupō*, which came 25 generations later. In the kōrero of Ngāti Kuri, the crew of the Kurahaupō lived on Rangitāhua for “many long and strenuous months” repairing their waka, using “Kaharoa,” a seal (kuri moana) net, providing one account of the origin of the name “Ngāti Kuri” (Ngāti Kuri 2013).

The name “Rangitāhua” itself refers to the sight of volcanic activity in the skies above the islands, with “Rangi” referring to the sky and “tahuhu” meaning “to set alight.” Other iwi throughout Aotearoa also hold connections to Rangitāhua through their own kōrero tuku iho. Ngāti Apa use the name “Rangitahuahua” for their wharenui at Whangaehu marae; in their kōrero, the crew of the *Kurahaupō* join onto the Aotea waka (He Oriori Mo Wharaurangi Waiata), symbolizing cooperation and Kotahitanga (Community Scoop 2012). In Taranaki, the name references a “day of feast” as a religious feast was held to bless their fleet's journey, a kōrero that emphasizes spiritual discipline and piety (Taipuhi and Tautahi 1900). The existence of diverse iwi kōrero tuku iho represents the mana and significance the moutere holds for Ngāti Kuri as ancestor and guardian that sheltered people and their waka, and for other iwi as lessons for future generations.

Despite these ancestral ties to Rangitāhua, for more than two centuries, the New Zealand government restricted Ngāti Kuri from accessing Rangitāhua while it increasingly became the focus of European expeditions and international whaling. During this time, natural history and archaeological taonga were collected and deposited in museums and collections, mostly in the Northern Hemisphere. In 2001, a group of Ngāti Kuri kaumātua voyaged to Rangitāhua and erected a pouwhenua, with words at its foot reading “Whakapūmau i to mātou rangatiratanga ki tenei whenua:” we entrench our rangatiratanga to this land (Pou Whenua 2001). This act represented a reclaiming of authority and connection to their ancestral territory.

A decade earlier, in 1991, Saana Waitai‐Murray of Ngāti Kuri had joined with other iwi leaders to lodge the Waitangi Tribunal claim (Wai262), with a rising concern about the lack of Māori consent and engagement in research that was occurring in their lands, the exploitation and commodification of taonga species, mātauranga Māori, and resources (Ringham 2023).

The Wai262 claim marked a significant shift in Waitangi Tribunal claims, becoming Aotearoa's first whole‐of‐government claim to move beyond historical treaty breaches, investigating contemporary treaty breaches that denied Indigenous ownership and control over intellectual property, cultural, and artistic works, and protection of relationships with taonga species (Waitangi Tribunal 2011). This groundbreaking claim set the stage for more direct collaborative approaches to research governance.

Translating these principles into practice, in 2015, the Ngāti Kuri Relationship Working Group (NKRWG) was founded to begin whakawhanaungatanga with research partners (Ringham 2023). These included Tāmaki Paenga Hira (Auckland Museum), Earth Sciences New Zealand (formerly NIWA), Bioeconomy Science Institute (former Manaaki Whenua Landcare Research), and Waipapa Taumata Rau (University of Auckland). The strategy was to position Ngāti Kuri as research leaders filling in knowledge gaps pertaining to iwi stories and taonga species, while their research allies could contribute scientific knowledge and support (Ringham 2023). Iwi members have been involved in data collection, scientific diving, scientific naming of species (e.g., rimurimu: D’archino et al. 2020; Nelson et al. 2023; Nelson et al. 2019), coauthorship, and hosting and attending national and global forums for ocean protection.

Building on this foundation, Te Mana o Rangitāhua (TMoR) emerged as a 5‐year (2020–2025) research program. TMoR develops transformative environmental wellbeing practices to enhance iwi and Aotearoa's ability to reconnect, reidentify, and restory the subtropical island ecosystem of Rangitāhua. The project is led by Ngāti Kuri in partnership with Tāmaki Paenga Hira, one of the oldest museums in Aotearoa (History of the Auckland Museum Institute ‐ Auckland War Memorial Museum).

Museums, herbaria, and other institutions holding natural history collections (NHC) have been described as “sentinel observatories of life on Earth” and “stewards of its future” (Krishtalka and Humphrey 2000). They are critical to the practice of taxonomy and as the repositories of vital reference specimens. NHC enable species to be studied, their distribution in space and time to be analyzed, and are fundamental resources for supporting biodiversity conservation (Johnson et al. 2011; Kharouba et al. 2019), and understanding the responses of flora and fauna to climate change (Graham et al. 2004; MacLean et al. 2019; Meineke et al. 2019; Meineke and Davies 2019).

While essential places of environmental knowledge, Museums and other NHC reverberate with colonial practices and worldviews. Museums have been complicit in colonial expansion and the silencing of Indigenous voices and knowledge systems (Wali and Collins 2023). The recognition of this complicity has led to growing discussion around the need for “decolonization” in the context of museum practice, here defined as “an ongoing process that involves restitution and rehumanization, notably through the sharing of knowledge and by encouraging mutual understanding” and “the distribution of museum's power to marginal groups” (Brulon Soares and Witcomb 2022). However, for decolonial museum practice to exist, the question of access to both material and immaterial collections needs to be addressed. “Data sovereignty,” the right for communities to hold decision‐making authority over their own data (Carroll et al. 2020; Poor 2022) and “repatriation,” the process of returning taonga to their original guardians (Skrydstrup 2009) are central actions that need to be upheld. However, access to the collections housed within institutions is controlled through systems that have, and often continue to, exclude Indigenous communities and people (Anderson 2005; Das and Lowe 2018). The degree of accessibility remains dependent on individual institutions’ willingness and capacity to share collections and their associated data (Edwards and Mead 2013; Wali and Collins 2023). This system perpetuates ongoing colonial domination of who has the collection viewing rights (Smith 2023).

The goal of the study described here was to strengthen Ngāti Kuri engagement with taonga and data from Rangitāhua—reclaiming knowledge and heritage—while providing a holistic documentation of the moutere's biodiversity and the connections between whenua and moana. Comprehensive stocktaking and provenance mapping was undertaken to understand the extent of collections from Rangitāhua and where the information and physical specimens are currently held around the world.

## 2 Materials and Methods

### 2.1 Expedition Data Collection

To create a timeline of past expeditions to Rangitāhua, a scoping data collection approach was followed between late 2021 and July 2022. Expedition data came mainly from secondary sources in the peer‐reviewed and grey literatures (Nickpour et al. 2022), while some additions were made based on the personal accounts of Ngāti Kuri knowledge holders and other researchers from the TMoR project team (Papaioannou et al. 2010). Grey literature consisted of government reports, newspaper and magazine articles, websites, photography, documentaries, and diary entries.

All peer‐reviewed literature was sought through the Scopus database using key terms commonly associated with Rangitāhua that covered the breadth of the topic (Arksey and O’Malley 2005). The search terms were: “Chanter Island” OR “Cheeseman Island” OR “Curtis Island” OR “Dayrell Island” OR “Denham Bay” OR “Egeria Rock” OR “Haszard Islet” OR “Herald Islet” OR “Kermadec Islands” OR “L’Esperance Rock” OR “Macauley Island” OR “Meyer Island” OR “Napier Islands” OR “Nugent Island” OR “Raoul Island” OR “Rangitāhua” OR “Roache Isle” OR “Sunday Island.” Additional peer‐reviewed articles that were missed were added based on the suggestions of the TMoR team. Grey literature was primarily sourced through Google searches of the same Rangitāhua search terms, with some additions from the collections of Tāmaki Paenga Hira.

For each expedition, the following description elements were recorded: expedition year/s; lead country; vessel name; expedition name and/or number; participating individuals; voyage purpose; landing status; locations visited; sampling depths reached; collections made; any other additional notes. We were unable to source information on one or more of the description elements for 64 of the 127 expeditions found. Validation of expedition dates was completed through triangulation of sources where repeating information was available (Maxwell 2012) and through review by TMoR members.

### 2.2 Expedition Categorization and Timeline Creation

To understand the historic trends of Rangitāhua expeditions, we created categories based on the reported main purpose of the expedition. In total, eight categories were created, shown in Table 1. The category and lead country of each expedition were encoded into a timeline visualization (Windhager et al. 2019).

**TABLE 1:** Categories applied to the historic expedition data.

Category	Definition
Mana whenua voyage	Voyages led by mana whenua, past or modern.
Early exploration	Earlier expeditions where the islands were first explored and mapped by international vessels (not mana whenua).
Biodiversity expedition	Expeditions with the primary purpose was the investigation of terrestrial or marine biodiversity.
Mapping/seamount/geology expedition	Expeditions with the primary purpose of mapping seafloor, exploring seamounts (nonbiodiversity focused), or other volcanology sampling.
Recreational voyage	Voyages for personal recreation.
Commercial expedition	Expeditions with the main purpose of commercial exploration.
Archaeology expedition	Expeditions primarily for the purpose of archaeology collection.
Routine inspection or placement for other reasons	Routine expeditions to deliver supplies or monitor conditions on Raoul Island. This includes war placement.

### 2.3 Specimen Data Collection

Curators, collection managers, and private collectors nominated by TMoR team members were contacted via email with an attached letter introducing our research. The letter explained the program's aim to establish a comprehensive database of existing knowledge and collections related to Rangitāhua, and it emphasized the global, multidisciplinary impact of the research database and its importance to Ngāti Kuri rangatiratanga. Recipients were requested to provide data on their collections, specimens, and observations from Rangitāhua and surrounding waters.

When a direct means of contact was unavailable, we approached staff through institutional websites or “general inquiries” channels. In some cases, the institutional dataset was readily available for download, and we did not need to contact any personnel. Whenever the database was not easily accessible or repeated follow‐ups (up to five attempts across 8 months) were unsuccessful, we obtained any available data relating to the institution's collections via GBIF (Global Biodiversity Information Facility) or any other available natural history data‐aggregating platform and considered the search complete.

To locate useful information from the publicly available datasets, we searched for the same keywords used for the expeditions stocktake; when the search engine allowed, the coordinates 26.390N to 32.039S and −179.074W to −175.941E were also used. Where no regional filtering was possible, all the hits for “New Zealand” and/or “Oceania” were downloaded in bulk and further filtered by searching the coordinates or keywords using R (Huettmann and Ickert‐Bond 2017; R Core Team 2023). To manage the data collection and track the status of communications with all the institutions, a supporting spreadsheet was developed and maintained daily (Table 2).

**TABLE 2:** Database structure for tracking communication and data collection progress with institutions holding natural history specimens and datasets.

Core identification	Institution name	Institution TypeMuseum/Herbarium/Research Institute/University/Private Collector	Continent	Country	
**Collection details**	Estimated count	Data type database/observations/preserved specimens/images/photo slides/bulletin/network	Research interest marine/botany/entomology/land vertebrates/archaeology/geology/paleology	Priority level1: Institutions in Oceania with physical specimens 2: Institutions in Oceania with digital records/institutions abroad with many physical specimens3: Institutions abroad with fewer specimens/digital records	
**Contact information**	Contact name	Contact email	Approach viadataset search/general inquiries/direct contact email/online form	Relationships holder	
**Communication tracking**	Date of first contact	Status of communicationletter sent/response received/data received/no data to add/database downloaded/none	Follow‐up action re‐send email/email another employee	Notes and links	Monthly review (e.g., review June 2024)data received/follow‐up next month/check dataset/done

### 2.4 Specimen Data Wrangling

The downloaded specimen data were maintained in a Microsoft Access database, through which tailored queries were used to tidy the data (convert the latitude and longitude, format the dates consistently, remove duplicates) prior to its compilation into a unified spreadsheet. When the discipline/research interest was not already provided by their institution/collection owner, specimens were divided into categories based on their kingdom/phylum/family/genus (e.g., Class = Aves would place a record into Ornithology).

To account for cases where a single record had a number of objects, which is not uncommon for biological specimens/observations, the object count was considered as 1 when not stated, and as 10, when the count was “multiple/many/several/pieces.” R packages were used to wrangle and visualize the data (Kahle and Wickham 2013; Wickham 2016; Neuwirth 2022; Wickham et al. 2023; Wickham and Bryan 2023).

The coordinates of each institution were obtained using Google maps’ API for R (Kahle and Wickham 2013). In cases where the records were kept by an online worldwide database, the coordinates of the data hosting institution were used instead (for example, Cornell Lab of Ornithology's coordinates were used for eBird).

### 2.5 Specimens and Expeditions Cross‐Referencing

A script on R implemented a comprehensive data linking and analysis workflow designed to match historical specimen records with their corresponding expedition voyages. The script incorporated two primary datasets: the specimen database with collection records, and the expedition register documenting historical voyages to Rangitāhua.

The core methodology employed a multitiered matching approach. First, the script standardized country names and expedition identifiers across both datasets to ensure consistent comparison. It then created composite matching keys combining two fields (country and year) to link specimens with their likely source expeditions. The matching process incorporated both exact and fuzzy matching techniques to account for historical inconsistencies in record keeping (Robinson 2020; Wickham 2023; Wickham and Henry 2023). The fuzzy matching component used the Jaro‐Winkler distance algorithm to identify similar expedition names that may have slight variations in spelling or formatting (Wang et al. 2017).

The script generated detailed matching statistics and produced several analytical outputs, including summaries of institutional participation in expeditions and the spatial–temporal distribution of specimen collections. Results were exported for further analysis and verification, providing a comprehensive dataset linking specimens to their probable expedition origins while documenting the certainty of each match. We divided the linking quality in three categories: high quality (cases in which expedition name and number were recorded); medium quality (cases in which no expedition name and numbers were recorded, but we had participating individuals and vessel name); and unlinked, in which not enough data was found in the expedition registry to enrich the collection data.

### 2.6 External Institutions Responsiveness’ of Data Requests

The dataset containing information on institutions holding Rangitāhua collections was imported into R and underwent systematic cleaning to standardize variables. Collection sizes were binned into four categories, ranging from small (<100,000 items) to very large (>10 million items), to facilitate comparative analysis. Likewise, institutional age (based on establishment year) was binned into four categories: <50 years; 50–99 years; 100–149 years; 150+ years.

The number of contact attempts, including initial letters and follow‐up nudges, was recorded as a measure of effort required to obtain responses. Success rates were calculated as the proportion of institutions providing successful responses out of the total contacted, both overall and stratified by institution type and collection characteristics. This classification system enabled quantitative assessment of factors influencing institutional cooperation and data accessibility across the global network of Rangitāhua collections.

### 2.7 Data Access and Data Sovereignty

The results and visualizations were reviewed by the TMoR team and other members of Ngāti Kuri at multiple in‐house and wider hui and wānanga. The datasets collected were provided to Ngāti Kuri and will be incorporated into their own knowledge portal.

## 3 Results

### 3.1 Expeditions Data

**FIGURE 1:**
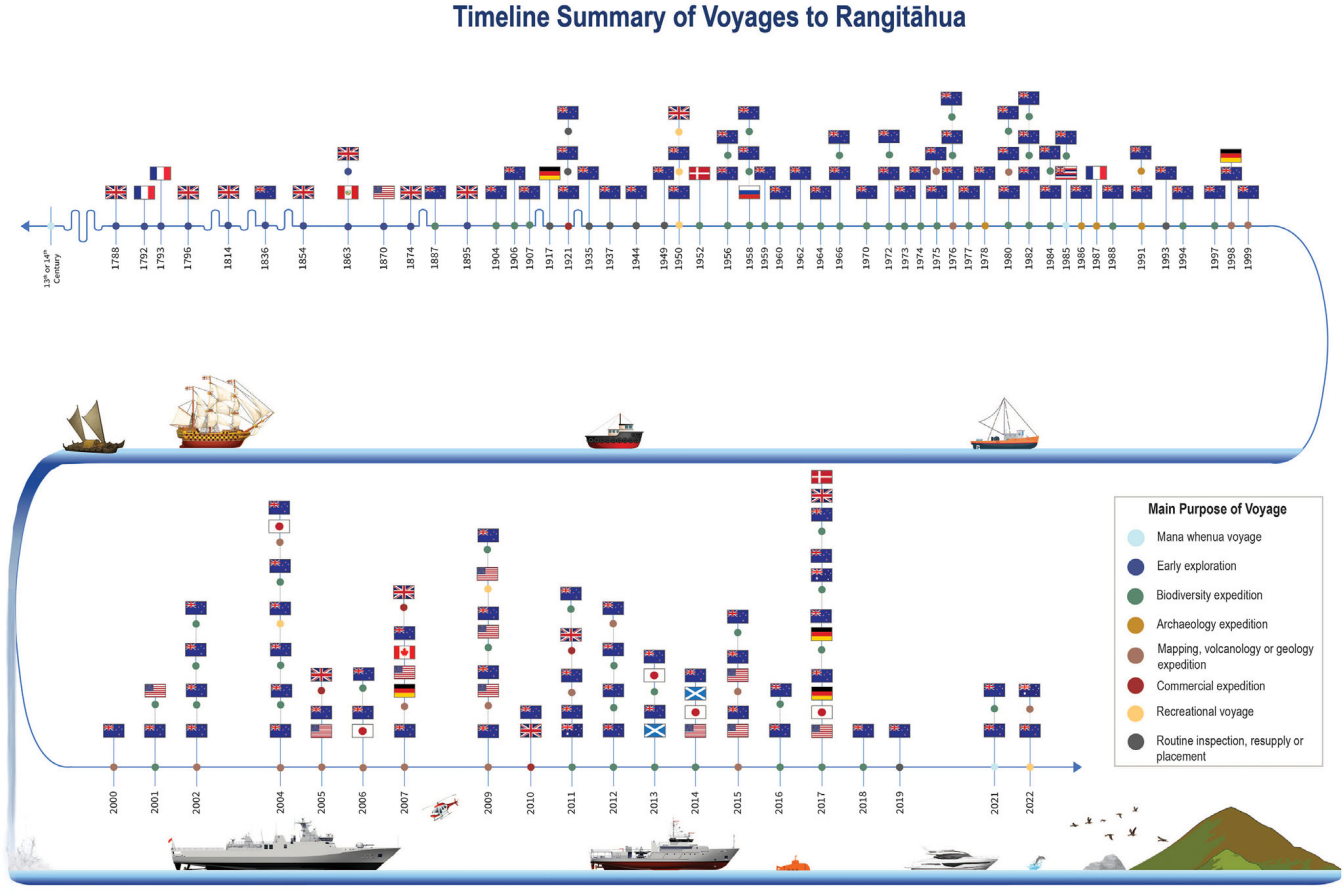
Chronological timeline of research voyages to Rangitāhua (1793–2022), categorized by expedition purpose.

In total, 127 expeditions were databased, with 124 having secondary source references, and the remaining three expeditions coming from verbal recounts of the TMoR team's knowledge only (Figure 1). Out of them, 95 (74%) have been led by or involved Aotearoa team members. Australia, Denmark, France, Germany, USA, Canada, Hawaii, Japan, Peru, Russia, Scotland, and England have all led or been involved in expeditions. Of all the expeditions listed, the most common categories were biodiversity and mapping (61) and seamount/geology (21) expeditions. As a stark contrast, only three expeditions (2%) were for the purposes of mana whenua voyaging.

Throughout the duration of TMoR, 12 voyages were successfully made to Rangitāhua. Five of them were led by mana whenua and/or had the full purpose of reconnecting with their tupuna. The other seven voyages were scientific expeditions that had at least one Ngāti Kuri member on board, with two of them being led by Ngāti Kuri.

### 3.2 Collection Data

**FIGURE 2:**
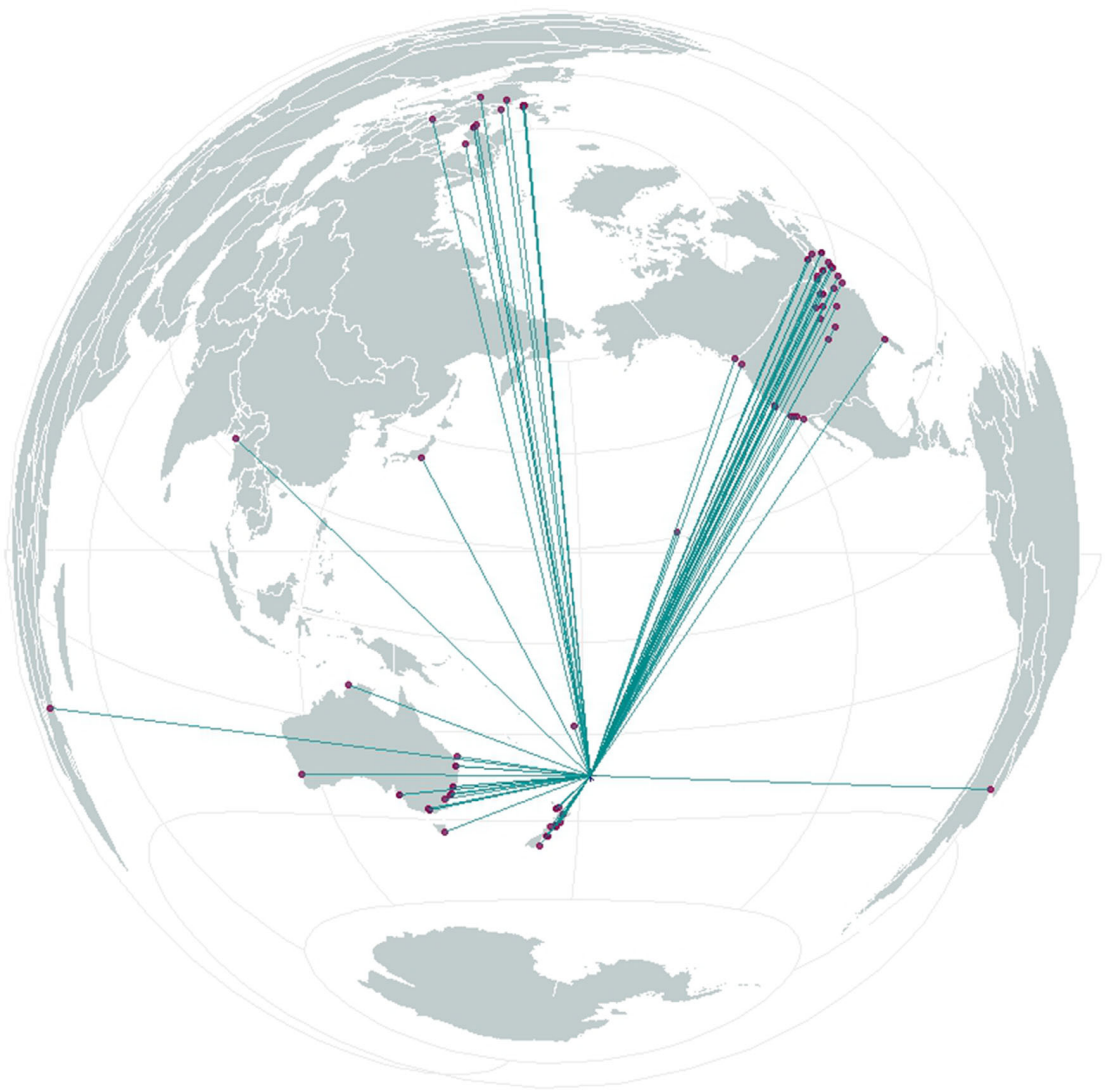
Global distribution of institutions (pink) holding specimens from Rangitāhua (blue).

In total, 40 804 records were downloaded, with over 1.73 million individual specimens and observations. The data were identified in 88 institutions worldwide, across five continents (Europe, the Americas, Oceania, Asia, and Africa) (Figure 2). Among the 40,804 records, 34,874 were physical (specimens, DNA, water samples), totaling 67,515 objects, an average of 1.93 specimens per record number. There were 5,930 digital (observation, photographs, sounds) records, totaling 1,664,696 individual observations, an average of 280 specimens per record number. For physical records, the maximum object count was 1353 for Phoridae (scuttle flies); for digital records, the maximum count was 1,000,000 for *Pterodroma cervicalis* (white‐necked petrel).

### 3.3 Expeditions and Collection Cross‐Referencing Data

**FIGURE 3:**
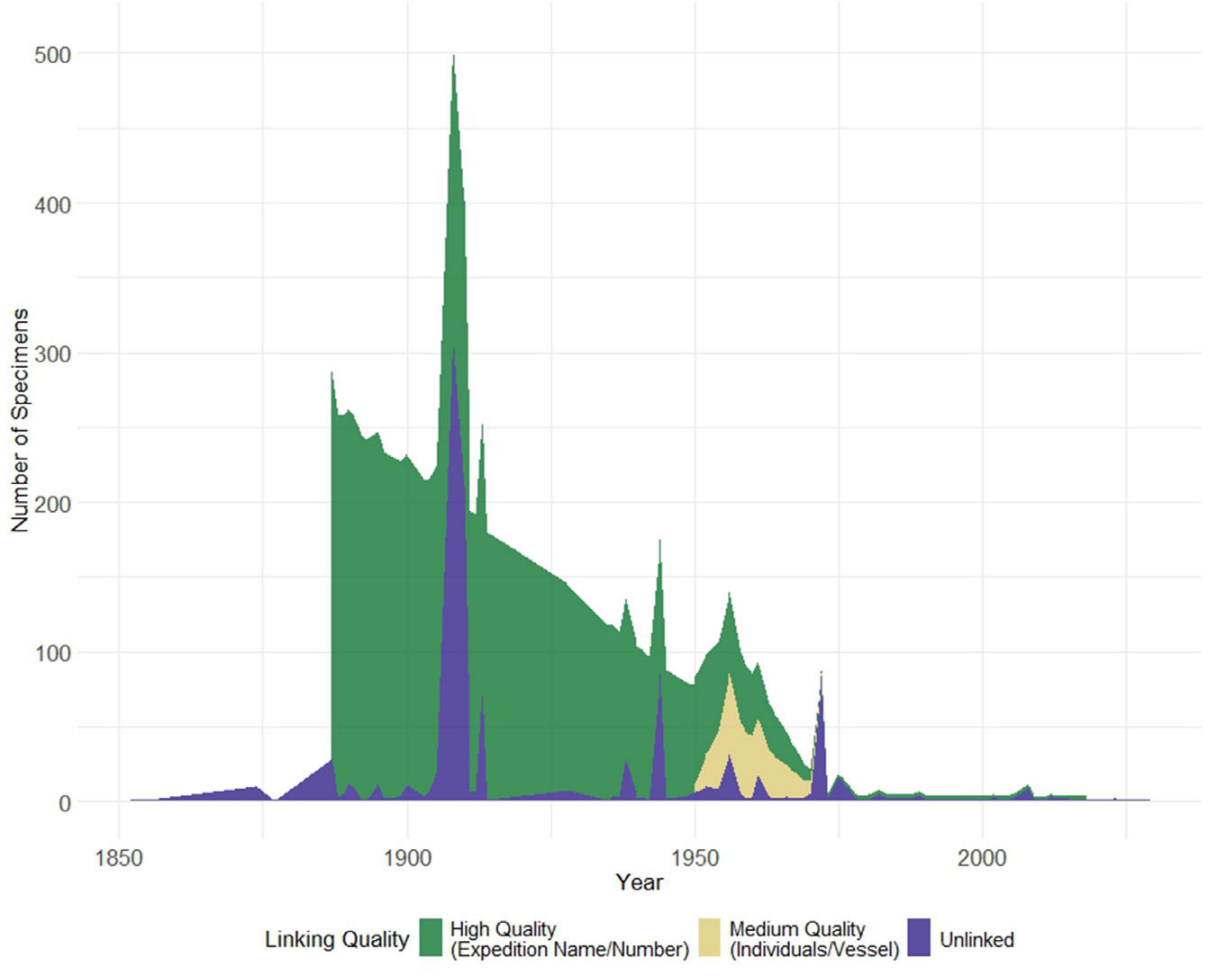
Assessment of the linking quality between museum specimens and associated expedition records following the cross‐referencing protocol.

The cross‐referencing of the collection and expeditions datasets provided different success rates across each decade (Figure 3). The earliest tracked voyage had 266 specimens successfully mapped, being transported by SS Stella to Tāmaki Paenga Hira in 1886. Another interesting result was the tracking of 174 records from Auckland Museum, Allan Herbarium, Canterbury Museum, Te Papa, and Otago Museum in a range of disciplines (Botany, Marine, Entomology, and Ornithology) in 1944 on a New Zealand Defense Forces voyage whose further details could not be sourced. We also noticed that recreational voyages, such as 1950s Lady Sterling and Huia expeditions, also involved the collection of specimens, which is the case for 24 specimens deposited at Te Papa and Tāmaki Paenga Hira. Another prolific year of collections was 1974, in which 1,923 specimens collected on board of RV *Tangaroa* were distributed to Tāmaki Paenga Hira, Allan Herbarium, Bioeconomy Sciences (formerly Manaaki Whenua), Earth Sciences New Zealand (formerly NIWA), and Te Papa.

Other leading countries had less successful linking rates; for instance, we were only able to track the provenance of three objects from the Museum and Art Gallery of the Northern Territory in Australia, coming from a 2022 voyage that researchers from the University of Tasmania joined. We identified five specimens deposited at Harvard University and Scripps Research Institute coming from RV Melville's voyage in 2001, and five specimens at Field Museum of Natural History and Harvard University collected in 2009 during The Wild Edge of the Pacific Expedition. Lastly, we were able to track 13 specimens at the Zoological Museum of Denmark that were transported through the 1952 voyage on board of Galathea.

### 3.4 Institutions’ Responsiveness

**FIGURE 4:**
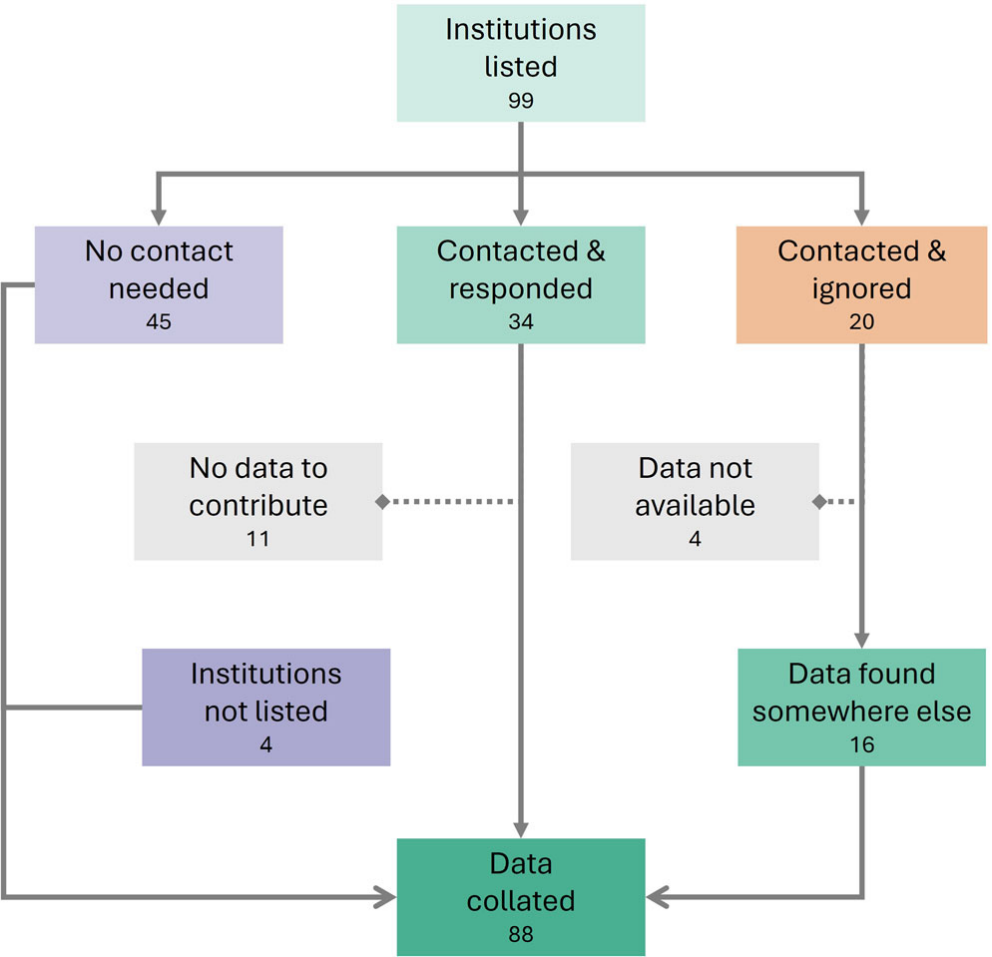
Flow diagram showing the data collection process from 99 initially listed institutions, resulting in the collation of information from 88 institutions through various contact and search strategies.

Our systematic approach to institutional data collection is detailed in Figure 4. The process revealed that nearly half of the institutions (45) required no contact as their data were publicly available on their “self‐service” portals. Of the other 54 institutions, 34 responded to contact attempts while 20 were unresponsive. Additionally, four institutions not initially listed were included as data pertaining to Rangitāhua was identified through aggregating knowledge portals.

**FIGURE 5:**
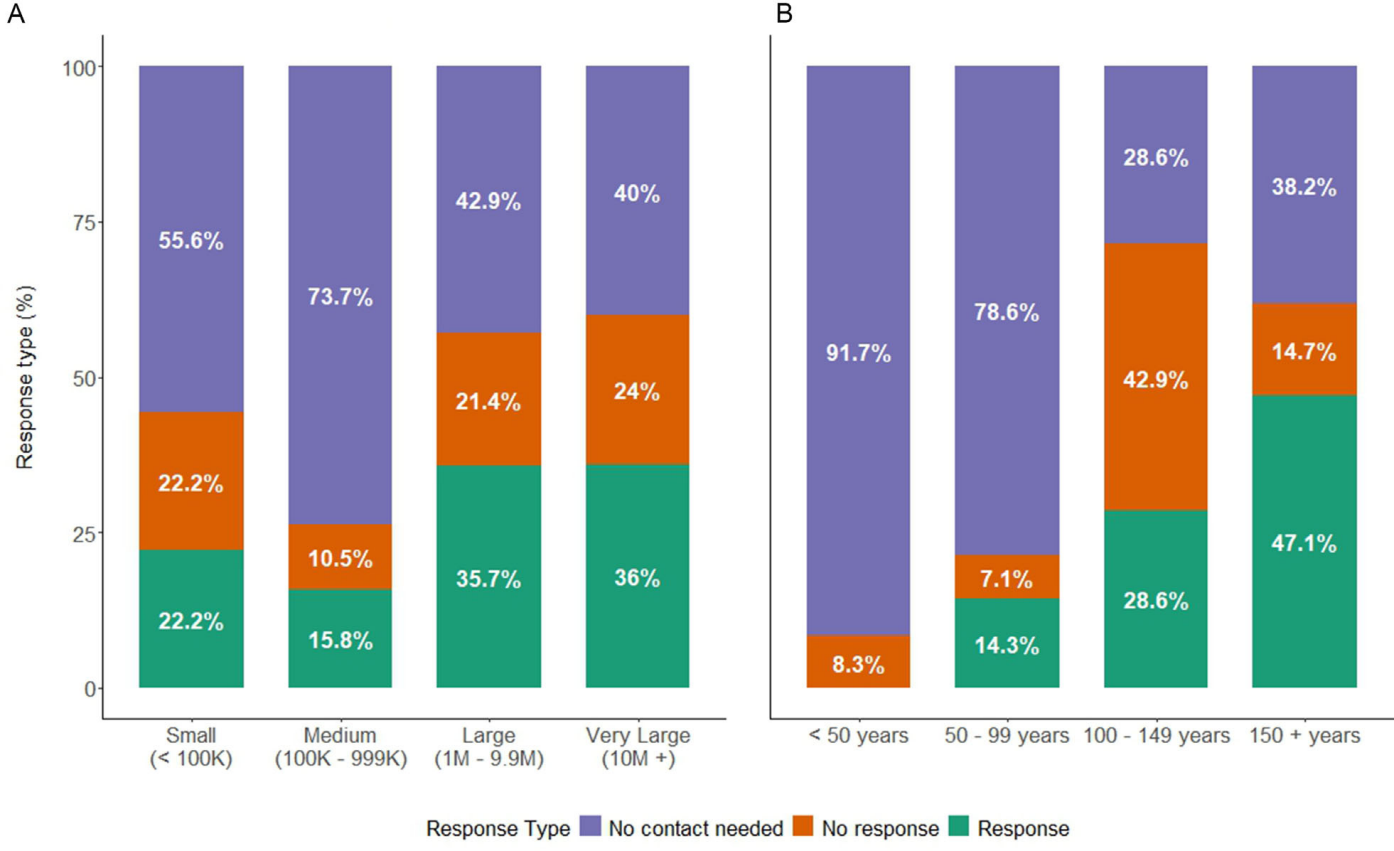
Response rates to specimen data requests by institutional collection size (A) and institutional age (B). Collection size and institutional age publicly available at institutional portals.

Regarding institutional responsiveness, we demonstrated that collection size seems to lightly influence institutional cooperation (Figure 5A), though the relationship seems to be less linear than with age (Figure 5B). Very large collections (10M+ items) showed moderate responsiveness at 36%, with large collections (1M–9.9M items) having a similar rate of 35.7%. Interestingly, small collections (<100K items) had a higher response rate than medium‐sized collections (100K–999K) with 22.2% and 15.8%, respectively.

We found that age played a more obvious role, with the oldest institutions (150+ years) showing the highest response rate at 47.1%, followed by institutions 100–149 years at 28.6% and 50–99 years old at 14.3% (Figure 5A). Notably, 91.7% of the youngest institutions (<50 years old) had superior self‐service infrastructure, which justified their lack of responses. Similarly, 78.6% of the 50–99 years old institutions had good self‐service portals, a sharp contrast with older institutions (100–149: 28.6% and 150 + years old: 38.2%).

In regards to contact attempts, correlation analysis revealed moderate positive relationships between collection size and contact attempts (*r* = 0.25) and between contact attempts and visitor/student numbers (*r* = 0.26), suggesting that larger, more visited institutions may require more persistence to obtain responses, possibly due to higher administrative burdens or more complex approval processes.

### 3.5 Method Limitations

Our data collection process had some limitations. Our compiling of expedition records was limited to information available through Scopus and Google databases, Tāmaki Paenga Hira collections, and TMoR team knowledge. Given the islands’ long expedition history, it is likely that some expeditions were missed. For instance, Ngāti Kuri holds a wealth of knowledge about their historic voyaging, but at the time of data collection, the iwi was still compiling this knowledge for themselves.

In our survey of institutional collection holdings, we relied on adequate specimen documentation. A number of institutions lack complete electronic records of the material in their collections, and in some cases, the inadequacy of other forms of record keeping may have precluded access to material.

Our data analysis approaches also introduced some limitations. We used fuzzy matching in our cross‐referencing of expedition and collection data due to quality issues in historical records, which may have led to some missed connections and false positives. Additionally, our analysis does not account for institutions that may have been willing to share data but lacked digital infrastructure or resources to respond effectively.

## 4 Discussion

The study represents a critical step toward decolonizing access to Rangitāhua's natural heritage. Through systematic tracking of all documented expeditions to Rangitāhua and comprehensive cataloging of specimens collected, we have created the first provenance map linking specimens to their collecting expeditions and the current housing institutions. Our research revealed the extent of extractive taonga collection that has happened at Rangitāhua over the centuries. The cataloging and mapping process also highlighted the unfortunate ongoing institutional barriers that perpetuate colonial control over NHCs.

The majority of research into decolonizing museum practice relates to ethnographic collections (Belsey 2019; O’brien 2019; Van Broekhoven 2019; Wali and Collins 2023), while NHCs have received less attention. The sheer volume and range of NHCs significantly outnumber those of any other museum discipline, yet the history of these collections—their cultural and social stories—is rarely documented (Das and Lowe 2018; Ashby 2023). NHCs from Rangitāhua have been collected and recorded in inherited systems, which remove the care and treatment Māori would give to the taonga species. Very rarely ingoa Māori are applied, with no kōrero giving meaning and history to the samples taken. There is a wealth of opportunities to uphold Indigenous data sovereignty within NHCs, if only it is made a priority (Pool 2016).

This project presented one such opportunity for institutions to engage in meaningful natural history data sharing with an Indigenous community that holds close ties to those collections. Our survey revealed a spectrum of institutional responses to our data requests, with most falling between two extremes. At one end, we received prompt and cooperative responses, while at the other, we received no response despite multiple contact attempts. The most common outcome fell between these poles: institutions either provided a delayed response (ranging from 3 weeks to several months) with a dataset extract, or maintained well‐developed, searchable databases that eliminated the need for direct personnel contact.

Our experience of receiving no responses to data access requests from 20 institutions demonstrates the data access restrictions faced by Ngāti Kuri—and, more broadly, Indigenous peoples—as they seek to access their own ancestral taonga. Whether this barrier is embedded gatekeeping (conscious or unconscious) on the part of institutions, or a consequence of inadequate resourcing, or a lack of recognition of the cultural importance of the collections they hold, the outcome is the same: a failure to uphold institutional stewardship responsibilities. This effectively renders the valuable knowledge contained within these collections unavailable to the very communities and researchers who would benefit from it the most (Johnson et al. 2023). The failure of so many institutions to respond raises fundamental questions about the goals of holding collections in the first place. What are specimens being preserved for? How can the original guardians of taonga access them if they do not know where they are? Can institutions effectively fulfill their responsibilities as guardians of collections if they do not acknowledge the cultural significance of the taonga contained within them? As stated by Hakiwai (2025), “Museums must be more than passive repositories that hold vast collections of objects and treasures of cultures and peoples throughout the world.”

Restoring sovereignty and relationship with taonga and Indigenous peoples can result in lasting, powerful benefits for the community. Stevens (2012) reflected on the process of Ngāi Tahu restoring sovereignty over Pounamu in the South Island during their settlement process, pointing to a local whakataukī, “for us and our children after us.” Taonga species hold knowledge, learnings, and gifts for the next generation; the extensive process we faced in assembling and acquiring information about the natural collections of Rangitāhua should be the last for this tūpuna moutere.

These benefits are equally as true for the data and information about taonga, as they are for the taonga themselves. As Hudson et al. (2023) state, reclaiming data sovereignty includes the rights of Indigenous people to “recover and/or repossess known/unknown data about their peoples, communities, ancestors, and nonhuman relations.” The use of the term “recover” is particularly apt in this definition, as the journey of Indigenous data sovereignty is not restricted to the independent gathering and control of data, but the possession of data previously taken. For Ngāti Kuri, these natural collections hold data that can enable research across many disciplines and inform iwi management practices while helping strengthen protection of Rangitāhua for future generations.

Improving Indigenous data sovereignty over NHCs not only has long‐lasting benefits for Indigenous communities but can also benefit the holding institutions themselves, creating reciprocal sharing channels to learn more about collections. If respectful relationships can be forged, where Indigenous peoples are provided the space to access and oversee the control of information about their taonga, they may feel comfortable sharing new knowledge about taonga origins, names, uses, and more with the holding institutions. However, this will only happen if NHC institutions begin to reflect upon their current barriers that ignore Indigenous rights over their own taonga and the data that is created about it.

The stocktake protocol that is described here provides a rich resource for future investigations and can serve as a blueprint for similar research initiatives that seek to begin the process of decolonizing NHC information in Aotearoa and beyond. TMoR has yielded critical information about Rangitāhua's biodiversity and changing conditions over time, while also exposing the complexity of navigating access to Indigenous heritage held globally. This work supports TMoR's and NKRWG's broader mission to restore Ngāti Kuri's mana whenua and reconnect the iwi with their ancestral knowledge, while providing a foundation for future repatriation efforts and the reclamation of Indigenous authority over their natural heritage.

The ongoing struggle to obtain access to centuries of natural materials and relevant data is a continuing issue for Indigenous people fighting for data sovereignty. This study into Rangitāhua's natural collections has constructed a pūrākau of its own, a history to be told through time that shows taonga taken in the past, access resisted in the present, and hope for a future where this does not continue.

## Nomenclature

**Māori kupu**
Hui
: meeting
Ingoa
: names
Iwi
: tribe
Kaitiaki
: guardian
Kaumātua
: respected tribal elders
Kōrero tuku iho
: stories passed down
Kōrero
: narrative, conversation
Kotahitanga
: unity or solidarity
Kupu
: words
Mana
: integrity, prestige, status, authority
Mana whenua
: tribal territorial authority
Marae
: complex of buildings that serve as sacred gathering places
Mātauranga
: knowledge
Moana
: ocean
Moutere
: island
Pakiwaitara
: stories of tupuna
Pounamu
: greenstone, jade
Pouwhenua
: carved territorial marker
Pūrākau
: stories
Puu Kaiao
: living protocol, culturally safety agreement
Rangatiratanga
: sovereignty
Rimurimu
: seaweed
Taiao
: environment
Taonga
: treasure
Tikanga
: protocols
Tohu
: environmental indicator
Tūpuna moutere
: ancestral islands
Tupuna
: ancestor
Tūpuna
: ancestors
Waka
: canoes
Wānanga
: deliberative workshops
Whakataukī
: Māori proverb
Whakawhanaungatanga
: building working relationships
Whenua
: land, placenta

## Funding

This study was supported by Ministry of Business, Innovation, & Employment (Grant AWMMU2001).

## Disclosure

The authors report there are no competing interests to declare.

## Data Availability Statement

The data that support the findings of this study are available in the supplementary material of this article.

## Conflicts of Interest

The authors declare no conflicts of interest.
